# Supplementary material for: Kasugamycin potentiates rifampicin and limits emergence of resistance in Mycobacterium tuberculosis by specifically decreasing mycobacterial mistranslation
Source: eLife. 2018 Aug 28;7:e36782. doi: 10.7554/eLife.36782 (PMC6160228; doi:10.7554/eLife.36782)
Supplement: Supplementary file 1. [file elife-36782-supp1.docx]

**Supplementary Table 1**

| Antibiotic | Minimum Inhibitory Concentration (MIC) (µg/ml) | | C_max_ / MIC^*^ |
| --- | --- | --- | --- |
|  | *M. smegmatis*-mc^2^-155 | *M. tuberculosis*-H37Rv |  |
| Rifampicin | 2.5 | 0.125 | N.D. |
| Kasugamycin | 2000 | 400 | 0.77 |
| Streptomycin | 0.5 | 0.5 | 7^†^ |

^*^ C_max_/MIC calculated from maximal plasma concentration of drug and the *in vitro* MIC of H37Rv.

^†^ The C_max_ for Streptomycin at the delivered dose (3mg/kg) was derived by linear regression of the PK measurements shown in Figure 2, Supplemental Figure 1.
