# Supplementary material for: Kasugamycin potentiates rifampicin and limits emergence of resistance in Mycobacterium tuberculosis by specifically decreasing mycobacterial mistranslation
Source: eLife. 2018 Aug 28;7:e36782. doi: 10.7554/eLife.36782 (PMC6160228; doi:10.7554/eLife.36782)
Supplement: Supplementary file 2. [file elife-36782-supp2.docx]

**Supplementary Table 2 Detailed statistical analysis of data presented in Fig. 2C**

| Statistical analysis of Fig. 2C | | |
| --- | --- | --- |
| Comparison | P values | |
|  | Lung pair | Spleen |
| No drug vs Strep | 0.1571 | 0.9674 |
| No drug vs Ksg | 0.0019 | 0.0322 |
| No drug vs Rif | < 0.0001 | 0.0007 |
| No drug vs Rif + Strep | < 0.0001 | < 0.0001 |
| No drug vs Rif + Ksg | < 0.0001 | < 0.0001 |
| Strep vs Ksg | 0.9774 | 0.6182 |
| Strep vs Rif | < 0.0001 | 0.0783 |
| Strep vs Rif + Strep | < 0.0001 | 0.0191 |
| Strep vs Rif + Ksg | < 0.0001 | < 0.0001 |
| Ksg vs Rif | < 0.0001 | 0.5831 |
| Ksg vs Rif + Strep | < 0.0001 | 0.2076 |
| Ksg vs Rif + Ksg | < 0.0001 | < 0.0001 |
| Rif vs Rif + Strep | 0.8850 | 0.9926 |
| Rif vs Rif + Ksg | 0.9081 | 0.0001 |
| Rif + Strep vs Rif + Ksg | 0.2690 | 0.0006 |
| Oneway ANOVA followed by Tukey’s multiple comparisons test | | |
