# Supplementary material for: Kasugamycin potentiates rifampicin and limits emergence of resistance in Mycobacterium tuberculosis by specifically decreasing mycobacterial mistranslation
Source: eLife. 2018 Aug 28;7:e36782. doi: 10.7554/eLife.36782 (PMC6160228; doi:10.7554/eLife.36782)
Supplement: Supplementary file 3. [file elife-36782-supp3.docx]

**Supplementary Table 3**

Oligonucleotides used in this work

| Primer Sequence | Purpose |
| --- | --- |
| 5´TTAATTAAGAAGGAGATATACATatgGTCTTCACCCTGGAGGACTTCGTCGGC-3´ | Cloning *nluc* F |
| 5´GTGCGAGCGCATCCTCGCCTGAGATATC-3’ | Cloning *nluc* R |
| 5´GCAACAAGATCATCAACGAGCGCCTCAT-3´ | SDM of D140N Nluc F |
| 5´ATGAGGCGCTCGTTGATGATCTTGTTGC-3´ | SDM of D140N Nluc R |
| 5´TTAATTAAGAAGGAGATATACATCATGTCGAAGGGCGAGG-3´ | Cloning *gfp* F |
| 5´GATATCCTAAGTCTACTTGTACAGCTCGTCCATGCC-3´ | Cloning *gfp* R |
| 5’GCTCTAGAACTAGTGTTTAAACTCTAGAAATATTGGAT-3’ | Cloning leaderless *Psmyc::mCherry* |
| 5’CAGTGCTATCCATTCCCAGAGCCTACTCGACCGAGCAC-3’ |  |
| 5’TAGGCTCTGGGAATGGATAGCACTGAGAGCGGCTCCACCGAG-3’ |  |
| 5’CCTTAATTAAAAAAGGCCATCCGTCAGGATGGCCTTCTCTAAACTGCTGCAGCGTAGTT-3’ |  |
| 5’CCATCGATAATATTGGATCGTCGGCACCGTCAC-3’ | Cloning *Psmyc::gfp* |
| 5’CCAAGCTTCTAAACTGCTGCAGCGTAGTTTTCGT-3’ |  |
| 5’CGGACTAGTACTAAACTGCTGCAGCGTAGTTTTCGTCGTTTGCTGCAGGCCTCTTGTACAGCTCGTCCATGCCGTG-3’ |  |
